# Androgen receptor regulates eIF5A2 expression and promotes prostate cancer metastasis via EMT

**DOI:** 10.1038/s41420-021-00764-x

**Published:** 2021-12-04

**Authors:** Yuancai Zheng, Ping Li, Hang Huang, Xueting Ye, Wei Chen, Guodong Xu, Fangyi Zhang

**Affiliations:** 1 Department of Urology, The First Affiliated Hospital of Wenzhou Medical University, Wenzhou, 325000 China; 2 Department of Cardiothoracic Surgery, The Affiliated Hospital, Ningbo Medical Center Lihuili Hospital, Ningbo University, Ningbo, Zhejiang 315041 China

**Keywords:** Cancer, Cell biology

## Abstract

Androgen receptor (AR) is an androgen-activated transcription factor of the nuclear receptor superfamily. AR plays a role in the development and progression of prostate cancer (PCa). However, the exact role of AR in PCa metastasis remains unclear. In the present study, we aimed to elucidate the function of AR in PCa. We found that eukaryotic translation initiation factor (EIF) 5A2, an elongation factor that induces epithelial-to-mesenchymal transition (EMT) in PCa cells, was significantly upregulated after 5α-dihydrotestosterone (DHT) stimulation and downregulated after anti‐androgen bicalutamide treatment in PCa cells with high AR expression, but not in cells with low AR expression. Moreover, eIF5A2 knockdown could eliminate DHT-induced invasion and migration of AR-positive PCa cells. DHT treatment decreased epithelial expression of E‐cadherin and β-catenin but increased the expression of the mesenchymal marker proteins Vimentin and N-cadherin. DHT therefore induced EMT, and knockdown of eIF5A2 inhibited DHT-induced EMT. Moreover, in vivo study, Luciferase signals from the lungs of the eIF5A2 plasmid group indicated higher metastasis ability, and the eIF5A2 siRNA group had lower metastasis ability. Our results suggest that AR positively regulates eIF5A2 expression in androgen-dependent cells, and stimulation of AR expression and signaling in prostate tumors promotes PCa metastasis by EMT induction and upregulation of eIF5A2.

## Introduction

Prostate cancer (PCa) is the most common cancer among men worldwide [1]. Androgens are essential to the development and progression of PCa [2], and androgen deprivation therapy (ADT) is currently the predominant treatment for locally advanced or metastatic PCa [3]. Molecular and cellular functions of androgens are mediated by the androgen receptor (AR), a vital regulator of androgen signaling. AR alters the expression of its downstream genes by interacting with coregulators, including activators and repressors, thereby regulating the transcription of androgen response genes, which play a crucial role in prostate cancer metastasis [4, 5]. Therefore, the identification and characterization of the androgen-responsive genes, which are crucial for hormone-stimulated cancer growth, could lead to the discovery and development of new therapeutic targets and more effective therapies.

Eukaryotic translation initiation factor 5A2 (eIF5A2), an isoform of eIF5A, plays an essential role in mRNA translation [6], and is an oncogene that regulates cell proliferation, invasion, metastasis, and cancer progression in several cancers [7–10]. Epithelial-to-mesenchymal transition (EMT) facilitates cancerous cell metastasis [11]. eIF5A2 regulates EMT in several cancers, and contributes to invasiveness and chemo-resistance of tumors [12–16]. EIF5A2 overexpression in prostate cancer cells therefore a potential prognostic predictive factor and therapeutic target [17]. However, the exact role of eIF5A2 in PCa progression is unclear.

Although the functions of androgen signaling in prostate cancer progression have been studied [2], there are conflicting views on the mechanisms of androgen-mediated EMT regulation. Several studies have reported that androgen could activate EMT and its effectors [18], while other have shown EMT activation caused by androgen signaling inhibition [19]. AR has been implicated as a negative regulator of EMT activation in PCa cells [20]. These conflicting data require further study in order to elucidate the role of androgen signaling in EMT regulation and PCa progression.

In this study, we aimed to determine the role of eIF5A2 and AR in the regulation of PCa metastasis, and identify the relationship between eIF5A2 and AR in the regulation of PCa metastasis.

## Results

### Androgen regulates the expression of eIF5A2 in PCA cells in an AR-dependent manner

**Fig. 1:**
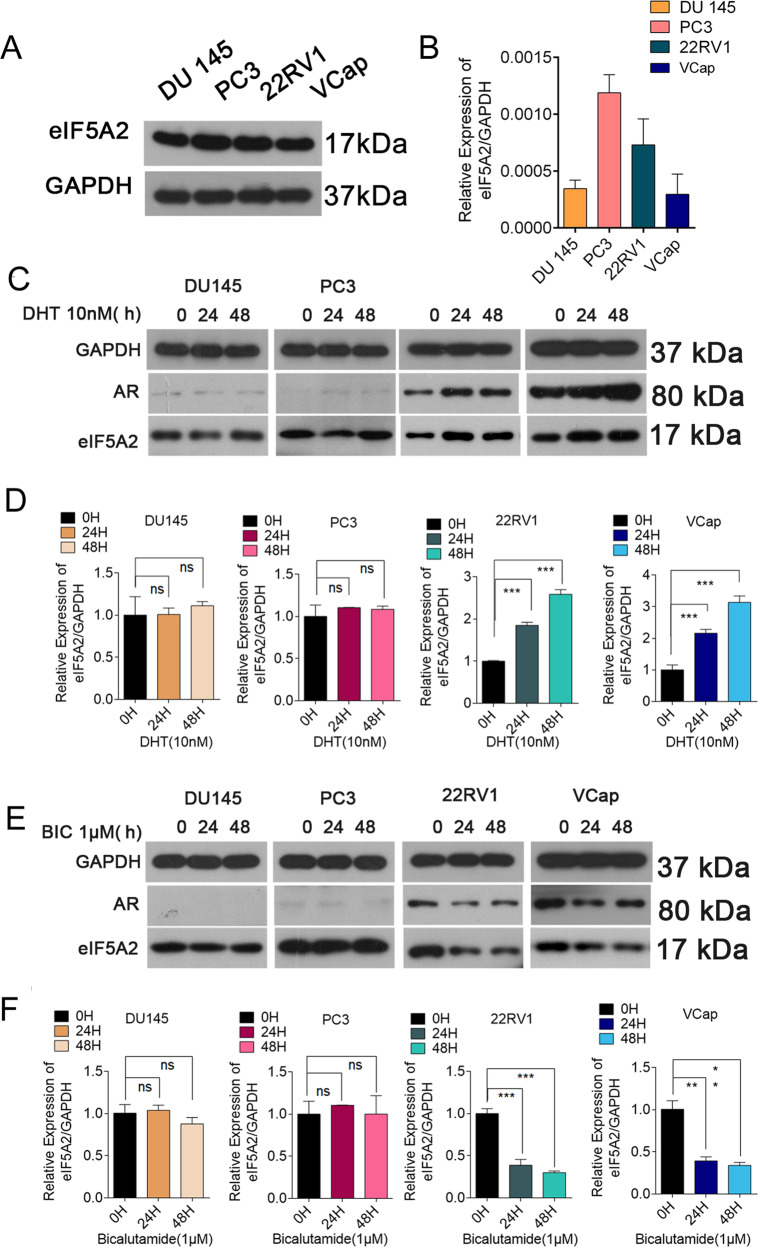
Androgen Regulates eIF5A2 expression in human prostate cancer cells in an AR-dependent manner. **A**, **B** Protein and mRNA levels of eIF5A2 in four PCa cell lines determined by western blotting and qRT-PCR, respectively. **C**, **D** Expression of eIF5A2 and AR in four PCa cell lines treated with DHT (10 nM) at 0, 24, 48 h. **E**, **F** Expression of eIF5A2 and AR in four PCa cell lines treated with bicalutamide (1 μM) at 0, 24, 48 h(NS, no significance, **P < 0.01,***P < 0.001 versus 0 h).

To study the interaction between eIF5A2 and AR, we first tested the expression of eIF5A2 in 4 PCa cell lines. As shown in Fig. 1A, B, the protein and mRNA levels of eIF5A2 were highest in PC3 cells. We then measured changes in eIF5A2 expression after androgen treatment. As shown in Fig.1C, D, DHT stimulation caused a time-dependent (0–48 h) increase in the protein and mRNA levels of eIF5A2 and AR in androgen-dependent PCa cells, but not in AI PCa cells. We then administered the AR inhibitor bicalutamide to confirm the relationship between eIF5A2 and AR. As expected, bicalutamide treatment caused a time-dependent decrease in the protein and mRNA levels of eIF5A2 and AR in androgen-dependent PCa cells, but not in AI PCa cells (Fig. 1E, F). We also performed AR siRNA transfection to determine whether androgen regulates the expression of eIF5A2 in PCa cells, and found that AR siRNA treatment could downregulate eIF5A2 expression in AD PCa cells, while DHT combined with AR siRNA did not further regulate eIF5A2 expression in AD PCa. Furthermore, compared with control group, whether treatment with AR siRNA alone or in combination with DHT did not change eIF5A2 expression in AI PCa cells (Fig. S1A). Thus, AR is necessary for the stimulatory function of androgens in eIF5A2 expression.

### EIF5A2 promotes the migration and invasion of PCA cells by EMT regulation

**Fig. 2:**
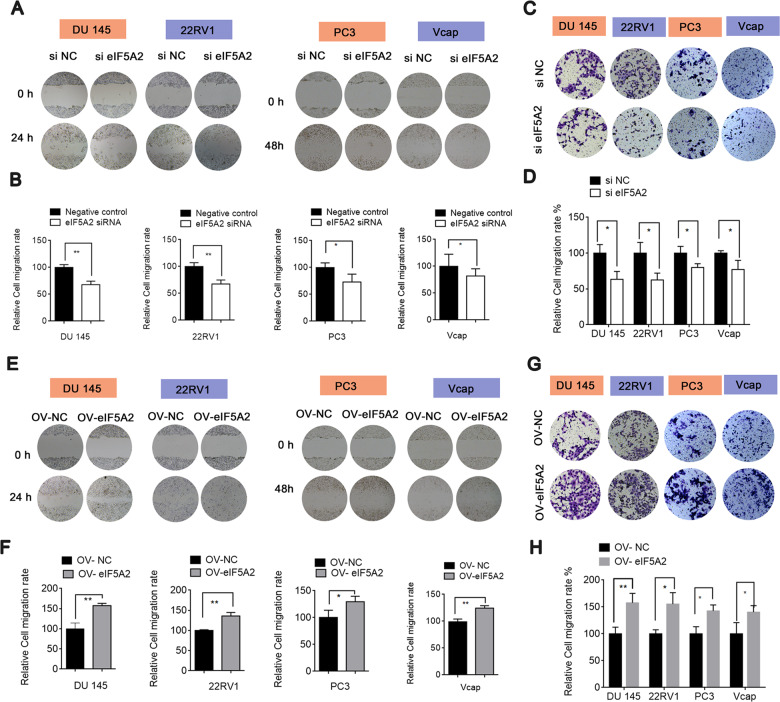
Effect of eIF5A2 on PCa cell migration and invasion. **A**, **B** Migration of DU145 and 22RV1 cells transfected with eIF5A2 siRNA or negative siRNA. **C**, **D** Invasion of PCa cells transfected with eIF5A2 siRNA or negative siRNA. **E**–**H** Migration and invasion of PCa cells transfected with eIF5A2 plasmid or negative plasmid. (*P < 0.05, **P < 0.01 versus negative control or plasmid NC).

Little is known about the role of eIF5A2 as a mediator of AR in PCa. We studied the function of eIF5A2 in PCa cells by eIF5A2 overexpression or knockdown. Three eIF5A2 siRNAs were screened and the one with the best interference efficiency was chosen for the further studies (Fig. S1B). EIF5A2 knockdown by siRNA could decrease PCa cell migration and invasion (Fig. 2A–D), while eIF5A2 overexpression by plasmid dramatically enhanced PCa cell migration and invasion (Fig. 2E–H).

**Fig. 3:**
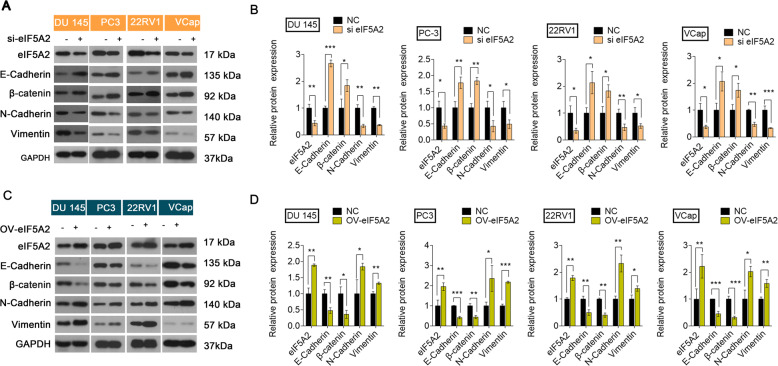
Effect of eIF5A2 on EMT. **A**, **B** Expression of EMT-related proteins in four PCa cell lines transfected with eIF5A2 siRNA or negative siRNA. **C**, **D** Expression of EMT-related proteins in four PCa cell lines transfected with eIF5A2 plasmid or negative plasmid.

EMT is involved in the invasion and metastasis of PCa cells [21, 22]. We therefore determined the effects of eIF5A2 on EMT in PCa cells. There is accumulating evidence that eIF5A2 could regulate invasion and migration in different cancers [23, 24]. We also found that eIF5A2 knockdown resulted in the upregulation of E‐cadherin and β-catenin, and downregulation of Vimentin and N-cadherin, thus inhibiting EMT (Fig. 3A, B). EIF5A2 overexpression showed the opposite effects, inducing EMT (Fig. 3C, D). These were consistent with the immunofluorescence results (Fig. S2A, B). The interfering efficiency of eIF5A2 siRNA and overexpression efficiency of eIF5A2 plasmid were confirmed by western blot (Fig. S1C, D). These results suggest that eIF5A2 expression could mediate PCa cell migration and invasion by promoting EMT.

### EIF5A2 knockdown attenuates androgen-induced cell migration and invasion

**Fig. 4:**
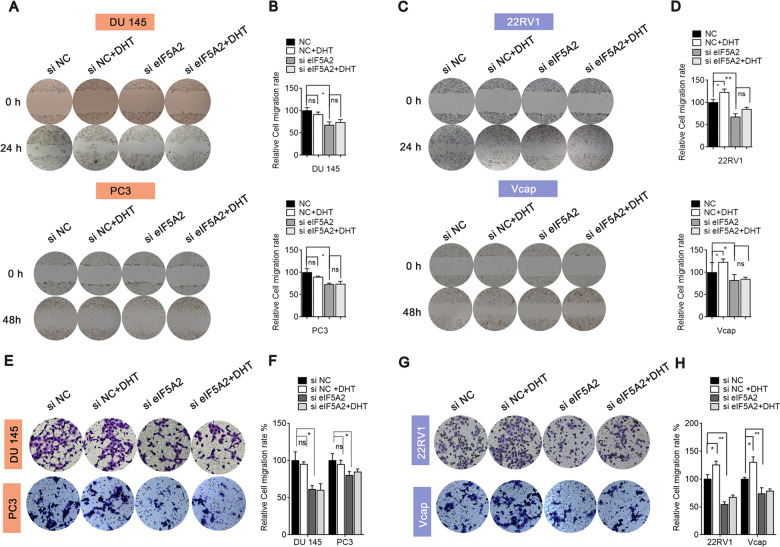
Effect of eIF5A2 on cell migration and invasion in the presence of DHT. **A**, **B** Migration of DU145 and PC3 cells transfected with eIF5A2 siRNA or negative siRNA in the presence or absence of DHT. **C**, **D** Migration of 22RV1 and Vcap cells transfected with eIF5A2 siRNA or negative siRNA in the presence or absence of DHT. **E**–**H** Invasion of PCa cells transfected with eIF5A2 siRNA or negative siRNA in the presence or absence of DHT. (NS no significance versus eIF5A2 siRNA; *P < 0.05, **P < 0.01).

To demonstrate that eIF5A2 is functionally relevant to AR, eIF5A2 siRNA or negative siRNA-transfected DU145, PC3 22RV1, and Vcap cells were cultured in medium with or without DHT, and cell migration and invasion were measured. As shown in Fig. 4A, B, DHT caused no differences in DU145 and PC3 cell migration compared with the control in the presence or absence of eIF5A2 knockdown. In 22RV1 and Vcap cells, DHT could enhance migration compared with the control; this effect was eliminated by eIF5A2 knockdown (Fig. 4C, D). Matrigel invasion assays also showed the same trend in PCa cells (Fig. 4E–H). We found that BIC, an inhibitor of AR, caused no differences in AI PCa DU145 cell migration compared to the control, and BIC combined with eIF5A2 plasmid also caused no differences in AI PCa DU145 cell migration compared to eIF5A2 plasmid group. But in AD PCa 22RV1 cells, BIC could decrease migration compared to the control, and furthermore it could reduce the increase of eIF5A2 plasmid-induced migration in 22RV cells (Fig. S3A). The invasion capacities of DU145 and 22RV1 cells, and EMT-related gene mRNA expression was studied following transfection with eIF5A2 plasmid with or without BIC treatment (Fig. S3B, C). The results were consistent with migration assay. Thus, eIF5A2 expression may contribute to AR-mediated PCa cell migration and invasion in the presence of androgens.

### Inhibition of eIF5A2 expression eliminates androgen-induced EMT

**Fig. 5:**
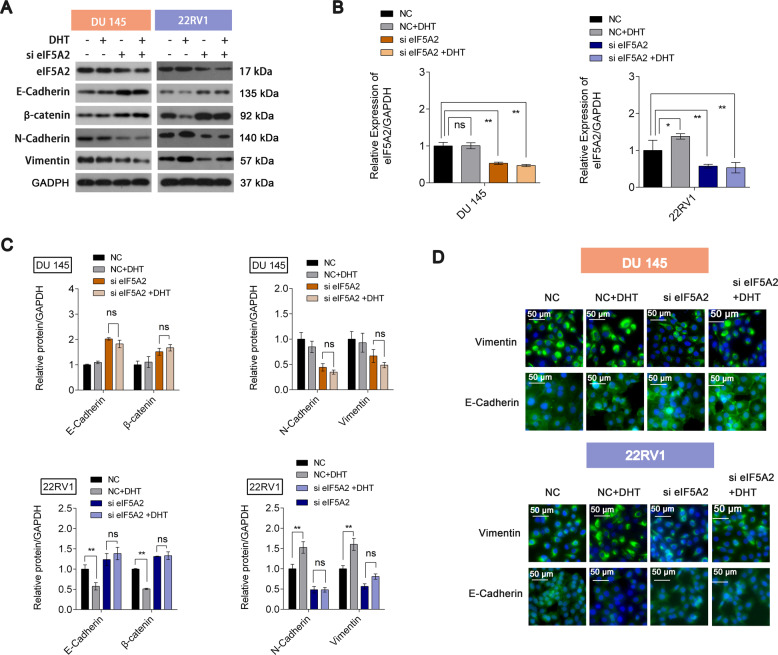
Effect of eIF5A2 on EMT in the presence of DHT. **A**, **B** Expression of EMT-related proteins in DU145 and 22RV1 cells transfected with eIF5A2 siRNA or negative siRNA in the presence or absence of DHT. **C** mRNA expression of eIF5A2 in DU145 and 22RV1 cells transfected with eIF5A2 siRNA or negative siRNA in the presence or absence of DHT. **D** Immunofluorescence staining of E-cadherin and Vimentin in DU145 and 22RV1 cells transfected with eIF5A2 siRNA or negative siRNA in the presence or absence of DHT.

Finally, we studied the role of DHT on EMT regulation and the role of eIF5A2 on DHT-mediated EMT regulation. DHT decreased the expression of E‐cadherin and β-catenin but increased the expression of Vimentin and N-cadherin, indicating DHT could induce EMT, in 22RV1 cells but not in DU145 cells. However, after eIF5A2 knockdown, EMT induction due to DHT was blocked in 22RV1 cells (Fig. 5A, B). Furthermore, BIC could upregulate the expression of E‐cadherin but downregulated Vimentin expression in 22RV1 cells but not in DU145 cells (Fig. S2B), and BIC also could reverse eIF5A2 plasmid-induced EMT in 22RV1 cells. eIF5A2 expression was confirmed in the four groups (Fig. 5C). Immunofluorescence results confirmed the results of western blot analysis (Fig. 5D). The metastasis-associated protein 1 (MTA1) gene was identified as a potential downstream target of eIF5A2 in CRC cells [10]. We therefore hypothesized a mechanism in which eIF5A2 regulates EMT-related factors by regulating MAT1 expression in PCa cells, and studied the effects of eIF5A2 siRNA and MAT1 plasmid on EMT-related protein expression. As shown in Figure. S4, eIF5A2 siRNA could reverse MAT1 plasmid-induced EMT (Fig. S4A, B). These results indicated that eIF5A2 could promote EMT of PCa through regulating MAT1. Furthermore, we determined the expression of EMT-transcription related factor, indicating that eIF5A2 siRNA treatment induced the changes of EMT-transcription factor TWIST1 and snail, furthermore, eIF5A2 siRNA combined DHT could downregulate TWIST1 and snail expression (Fig. S5).

### EIF5A2 regulates metastasis in vivo

**Fig. 6:**
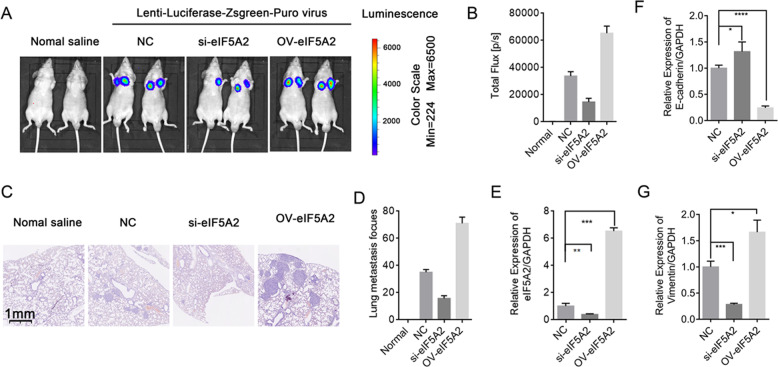
EIF5A2 regulates metastasis in vivo. **A**, **B** Luciferase expression in intrahepatic tumors in different treatment groups (normal saline, NC, eIF5A2 mimics, eIF5A2 inhibitor). **C**, **D** Images showing HE staining of lung tissues. **E**–**G** qRT-PCR confirmed the expression of eIF5A2, E-cadherin, and Vimentin expression after transfection with eIF5A2 siRNA or plasmid.

To elucidate the effect of eIF5A2 on PC metastasis in vivo, we transfected 22RV1 cells with Lenti-Luciferase-Zsgreen-Puro and then with NC, eIF5A2 siRNA, or eIF5A2 plasmid and observed lung metastasis in vivo. Luciferase signals from the lungs of the eIF5A2 plasmid group indicated higher metastasis ability, and the eIF5A2 siRNA group had lower metastasis ability compared with the control group at 4 weeks after injection (Fig. 6A, B). H&E analysis also showed that there was more metastasis in the lung after transfection with eIF5A2 plasmid (Fig. 6C, D). Compared with the NC group, the level of eIF5A2 and Vimentin was downregulated, the expression of E-cadherin was increased in eIF5A2 siRNA group, while eIF5A2 and Vimentin was upregulated, the expression of E-cadherin was reduced in eIF5A2 plasmid group by qRT-PCR(Fig. 6E–G).

## Discussion

The study of androgen responsive genes is important to obtain novel insights into the molecular mechanisms underlying androgen induced invasion, migration, and progression of prostate cancer cells [25, 26]. In this study, we identified eIF5A2 as an androgen responsive gene. Androgen stimulation could induce the upregulation of eIF5A2 in AR-positive PCa cells. This phenotype was not observed in AR-negative PCA cells. Thus, our results demonstrated that eIF5A2 expression was stimulated by androgen in an AR-dependent manner in PCa.

EIF5A2 belongs to the eIF5A gene family located on human chromosome 3q26.2, [27]. EIF5A2 mRNA is upregulated in several human tumor cell types such as pancreatic ductal adenocarcinoma, hepatocellular cancer, lung cancer, colorectal cancer, and gastric cancer, indicating that eIF5A2 is a potential oncogene [13, 28–31]. EIF5A2 regulates invasion and migration via regulation of EMT in various cancer cells [23, 24]. However, the function of eIF5A2 in PCa cells, and the interaction between eIF5A2 and androgens remains unclear. In this study, we demonstrated that eIF5A2 could promote the migration and invasion of PCa cells by inducing EMT changes. We also found that eIF5A2 siRNA could reduce lung metastasis in vivo. eIF5A2 siRNA could downregulated the level of eIF5A2 and Vimentin, upregulate the expression of E-cadherin group, while transfection with eIF5A2 have the opposite effect in vivo tissue. It has reported that MTA1 was identified as a potential downstream target of eIF5A2 in CRC cells [10], and further reveal the mechanism in which eIF5A2 regulates EMT-related factors in PCa cells. We co-transfected eIF5A2 siRNA and MAT1 plasmid to PCa cells and demonstrated that eIF5A2 siRNA could reverse MAT1 plasmid-induced EMT, indicated that eIF5A2 may regulate EMT-related factors via MAT1 in PCa cells.

The spread of cancer cells is considered to be a key step in tumor progression, similar to the epithelial–mesenchymal (EMT) transition observed during embryonic development. EMT is a process in which epithelial cells gradually lose cell–cell contact and acquire the movement and migration characteristics of mesenchymal cells. This transition is coordinated by the activation of transcription factor (EMT-TFs) of the snail and TWIST families, which directly inhibit a large number of epithelial marker genes involved in cell adhesion and polarity. Our findings also indicated that eIF5A2 siRNA treatment induced the changes of EMT-transcription factor TWIST1 and snail, furthermore, eIF5A2 siRNA combined DHT could downregulate TWIST1 and snail expression. EMT is closely related to AR signaling and is responsible for metastasis in PCa cells [19]. Our findings showed that androgen stimulation could induce the EMT phenotype in AR-positive PCa cells, but not in AR-negative PCa cells. These results are contradictory, since AR is a downstream factor of androgen stimulation. A possible explanation for this observation is that androgen exerts its function through AR-dependent pathways. To our knowledge, this is the first study showing that eIF5A2 reversed androgen-induced EMT in PCa cells, which was likely dependent on the presence of AR.

In conclusion, our results provide preliminary evidence of AR-mediated regulation of eIF5A2 expression in PCa cell metastasis via regulation of the EMT pathway. Strategies targeting AR/eIF5A2 axis could prove useful for targeting metastasis in PCa cells.

## Method and materials

### Cell culture and reagents

The androgen-dependent (AD) PCa cell lines 22RV1 and Vcap were purchased from the American type culture collection (Manassas, VA, USA), and the androgen-independent (AI) PCa cell lines, PC3 and DU145, were obtained from the cell bank of the Chinese academy of sciences (Shanghai, China). The cell lines were cultured in RPMI-1640 media (Invitrogen, Carlsbad, CA), supplemented with 10% fetal bovine serum (FBS, Invitrogen) at 37 °C in a humidified atmosphere with 5% CO_2_. 5α-dihydrotestosterone (DHT) and bicalutamide (BIC) were obtained from Sigma-Aldrich (St. Louis, MO, USA).

### Transfection

Cells were seeded in 6‐well plates at a density of 2 × 10^5^ cells, and cultured in complete medium for 18–24 h. After plating overnight, PCa cells were subjected to siRNA knockdown using eIF5A2 or negative control siRNAs (GenePharma, Shanghai, China). Lipofectamine2000 (Invitrogen) was used for siRNA knockdown according to the manufacturer’s instructions. For plasmid overexpression, PCa cells were plated overnight, and transfected with eIF5A2 (Genechem, Shanghai, China) or control vector (pCMV6‐Entry).

### RNA extraction and quantitative real-time PCR

Total RNA was isolated from treated cells by using TRIzol reagent (Invitrogen, Waltham, MA, USA) according to manufacturers’ instructions. RT-PCR was performed using the PrimeScript RT reagent kit (Takara Bio, Dalian, China) and *Premix Ex Taq*™ II qRT-PCR kit (TaKaRa) according to the manufacturers’ protocols. GAPDH was used as an internal control. The expression of eIF5A2 was analyzed using the comparative 2^−ΔΔCt^ method. The primers used were as follows:

eIF5A2:

Forward :5ʹ-GCAGACGAAAUUGAUUUCATT-3ʹ

Reverse :5ʹ-UGAAAUCAAUUUCGUCUGCTT-3ʹ;

GAPDH:

Forward 5ʹ-CGGAGTCAACGGATTTGGTCGTAT-3ʹ

Reverse 5ʹ-AGCCTTCTCCATGGTGGTGAAGAC-3ʹ

### Western blot analysis

Cells were lysed in RIPA lysis buffer (Beyotime, Shanghai, China). Protein concentrations were assayed using a BCA Protein Assay Kit (Pierce; Thermo Fisher Scientific, Inc.) with bovine serum albumin (Invitrogen) as the standard. 10% SDS-PAGE was used to separate equal amounts of proteins, which were transferred to PVDF membranes. The membranes were blocked with 5% (W/V) nonfat-dry milk in TBST for 1 h at room temperature, and incubated overnight at 4 °C with primary antibodies against eIF5A2 (1:1000; Abcam, Cambridge, UK) and GAPDH (1:1000; Abcam), and an EMT antibody sampler kit (1:1000; CST, Danvers, MA). After washing three times with TBST, the membrane was incubated for 2 h at room temperature with peroxidase-conjugated secondary antibodies (1:2000; Abcam). The protein bands were developed using enhanced chemiluminescence detection reagents (GE Healthcare, Chicago, IL, USA) according to the manufacturer’s protocol.

### Transwell invasion and wound healing assays

Cell invasion assay was performed using Transwells (Corning, NY). PCa cells were treated with trypsin and collected 48 h after transfection. 1 × 10^5^ cells were suspended in serum-free RMPI-1640 and inoculated into transwell inserts coated with matrigel (BD Bioscience, Bedford, MA). RMPI-1640 complete medium was then placed in the lower compartment. After 48 h, the cells were fixed and stained. The number of cells crossing the bottom surface of the membrane was counted and photographed. All experiments were carried out in triplicate. For the wound healing assay, treated cells seeded in 6-well plates at 3 × 10^5^ were “wounded” using a 200 μl micropipette tip when the cell confluence reached 90%. Images were captured at 0 and 24 h time points at different regions of the wound.

### Immunofluorescence staining

2 × 10^5^ PCa cells were cultured on glass slides in 24-well plates. After 24 h, cells were fixed in cold 4% paraformaldehyde, permeabilized in 0.1% Triton X-100, blocked with 1% BSA for 1 h, incubated with FITC-conjugated (Green) antibodies against E-cadherin (Abcam, 1:100) or Vimentin (CST, 1:50) overnight at 4 °C. DAPI was used to label nuclei. Fluorescent sections were observed and photographed under confocal fluorescence microscopy.

### Lung metastasis analysis in nude mice

The Lenti-Luciferase-Zsgreen-Puro virus (Hangzhou Shiyu Biotechnology Co. Ltd, Hangzhou, China) was transfected into a six-well plate of 22RV1 cells. Stable transfection was detected using 10 μg/ml of puromycin and fluorescence microscopy. A sufficient quantity of cells was prepared for transfection with NC, LV-eIF5A2 siRNA, and LV-eIF5A2 plasmid. Female BALB/c nu/nu mice (5–6 weeks old) were randomly divided into four groups (*n* = 8 per group): Normal saline, NC, eIF5A2 siRNA, and eIF5A2 plasmid. For lung metastasis analysis, 2 × 106 cells/mouse cells were injected into the tail vein of each nude mouse. Animals were weighed on alternate days to observe changes in body weight. Four weeks later, mice were intraperitoneally injected with D-luciferin, anesthetized with isoflurane, and photographed using an IVIS imaging system. The lungs were removed and fixed with 10% formalin. Subsequently, consecutive tissue sections were made from each block of the lung, and subjected to hematoxylin-eosin (H&E) staining according to the manufacturer’s protocol.

### Statistical analysis

Data were represented as mean ± SD from three independent experiments. Student’s unpaired *t*-test was used for two-group comparisons. A *P*-value of <0.05 was considered significant.

## Supplementary information

Supplementary Files

Authorship

## Data Availability

We declare that all data supporting the conclusions of the study.

## References

[1] Siegel RL, Miller KD, Jemal A (2017). Cancer statistics, 2017. CA Cancer J Clin.

[2] Zhou Y, Bolton EC, Jones JO (2015). Androgens and androgen receptor signaling in prostate tumorigenesis. J Mol Endocrinol.

[3] Martin PL, Yin JJ, Seng V, Casey O, Corey E, Morrissey C (2017). Androgen deprivation leads to increased carbohydrate metabolism and hexokinase 2-mediated survival in Pten/Tp53-deficient prostate cancer. Oncogene.

[4] Imamura Y, Sadar MD (2016). Androgen receptor targeted therapies in castration-resistant prostate cancer: bench to clinic. Int J Urol.

[5] Ko CJ, Huang CC, Lin HY, Juan CP, Lan SW, Shyu HY (2015). Androgen-induced TMPRSS2 activates matriptase and promotes extracellular matrix degradation, prostate cancer cell invasion, tumor growth, and metastasis. Cancer Res.

[6] Clement PM, Johansson HE, Wolff EC, Park MH (2006). Differential expression of eIF5A-1 and eIF5A-2 in human cancer cells. FEBS J.

[7] Liu Y, Du F, Chen W, Yao M, Lv K, Fu P (2015). EIF5A2 is a novel chemoresistance gene in breast cancer. Breast Cancer.

[8] Wei JH, Cao JZ, Zhang D, Liao B, Zhong WM, Lu J (2014). EIF5A2 predicts outcome in localised invasive bladder cancer and promotes bladder cancer cell aggressiveness in vitro and in vivo. Br J Cancer.

[9] Lou B, Fan J, Wang K, Chen W, Zhou X, Zhang J (2013). N1-guanyl-1,7-diaminoheptane (GC7) enhances the therapeutic efficacy of doxorubicin by inhibiting activation of eukaryotic translation initiation factor 5A2 (eIF5A2) and preventing the epithelial-mesenchymal transition in hepatocellular carcinoma cells. Exp Cell Res.

[10] Zhu W, Cai MY, Tong ZT, Dong SS, Mai SJ, Liao YJ (2012). Overexpression of EIF5A2 promotes colorectal carcinoma cell aggressiveness by upregulating MTA1 through C-myc to induce epithelial-mesenchymaltransition. Gut.

[11] Santamaria PG, Moreno-Bueno G, Portillo F, Cano A (2017). EMT: present and future in clinical oncology. Mol Oncol.

[12] Pan Q, Sun L, Zheng D, Li N, Shi H, Song J (2018). MicroRNA-9 enhanced cisplatin sensitivity in nonsmall cell lung cancer cells by regulating eukaryotic translation initiation factor 5A2. Biomed Res Int.

[13] Sun J, Xu Z, Lv H, Wang Y, Wang L, Ni Y (2018). eIF5A2 regulates the resistance of gastric cancer cells to cisplatin via induction of EMT. Am J Transl Res.

[14] Wang X, Jin Y, Zhang H, Huang X, Zhang Y, Zhu J (2018). MicroRNA-599 inhibits metastasis and epithelial-mesenchymal transition via targeting EIF5A2 in gastric cancer. Biomed Pharmacother.

[15] Xu G, Shao G, Pan Q, Sun L, Zheng D, Li M (2017). MicroRNA-9 regulates non-small cell lung cancer cell invasion and migration by targeting eukaryotic translation initiation factor 5A2. Am J Transl Res.

[16] Yang J, Yu H, Shen M, Wei W, Xia L, Zhao P (2014). N1-guanyl-1,7-diaminoheptane sensitizes bladder cancer cells to doxorubicin by preventing epithelial-mesenchymal transition through inhibition of eukaryotic translation initiation factor 5A2 activation. Cancer Sci.

[17] Lu J, Zhao HW, Chen Y, Wei JH, Chen ZH, Feng ZH (2019). Eukaryotic translation initiation factor 5A2 is highly expressed in prostate cancer and predicts poor prognosis. Exp Ther Med.

[18] Nakazawa M, Kyprianou N (2017). Epithelial-mesenchymal-transition regulators in prostate cancer: androgens and beyond. J Steroid Biochem Mol Biol.

[19] Nouri M, Ratther E, Stylianou N, Nelson CC, Hollier BG, Williams ED (2014). Androgen-targeted therapy-induced epithelial mesenchymal plasticity and neuroendocrine transdifferentiation in prostate cancer: an opportunity for intervention. Front Oncol.

[20] Izumi K, Fang LY, Mizokami A, Namiki M, Li L, Lin WJ (2013). Targeting the androgen receptor with siRNA promotes prostate cancer metastasis through enhanced macrophage recruitment via CCL2/CCR2-induced STAT3 activation. EMBO Mol Med.

[21] Hao H, Wang L, Chen H, Xie L, Bai T, Liu H (2017). YKL-40 promotes the migration and invasion of prostate cancer cells by regulating epithelial mesenchymal transition. Am J Transl Res.

[22] Chen L, Cao R, Wang G, Yuan L, Qian G, Guo Z (2017). Downregulation of TRPM7 suppressed migration and invasion by regulating epithelial-mesenchymal transition in prostate cancer cells. Med Oncol.

[23] Zhou X, Xu M, Guo Y, Ye L, Long L, Wang H (2018). MicroRNA-588 regulates invasion, migration and epithelial-mesenchymal transition via targeting EIF5A2 pathway in gastric cancer. Cancer Manag Res.

[24] Xu GD, Shi XB, Sun LB, Zhou QY, Zheng DW, Shi HS (2013). Down-regulation of eIF5A-2 prevents epithelial-mesenchymal transition in non-small-cell lung cancer cells. J Zhejiang Univ Sci B.

[25] Takayama K, Inoue S (2013). Transcriptional network of androgen receptor in prostate cancer progression. Int J Urol.

[26] Koochekpour S (2010). Androgen receptor signaling and mutations in prostate cancer. Asian J Androl.

[27] Clement PM, Henderson CA, Jenkins ZA, Smit-McBride Z, Wolff EC, Hershey JW (2003). Identification and characterization of eukaryotic initiation factor 5A-2. Eur J Biochem.

[28] Fujimura K, Wright T, Strnadel J, Kaushal S, Metildi C, Lowy AM (2014). A hypusine-eIF5A-PEAK1 switch regulates the pathogenesis of pancreatic cancer. Cancer Res.

[29] Tu C, Chen W, Wang S, Tan W, Guo J, Shao C, et al. MicroRNA-383 inhibits doxorubicin resistance in hepatocellular carcinoma by targeting eukaryotic translation initiation factor 5A2. J Cell Mol Med. 2019;23:7190–99. 10.1111/jcmm.14197 PMC6815770 30801960

[30] He LR, Zhao HY, Li BK, Liu YH, Liu MZ, Guan XY (2011). Overexpression of eIF5A-2 is an adverse prognostic marker of survival in stage I non-small cell lung cancer patients. Int J Cancer.

[31] Deng B, Wang B, Fang J, Zhu X, Cao Z, Lin Q (2016). MiRNA-203 suppresses cell proliferation, migration and invasion in colorectal cancer via targeting of EIF5A2. Sci Rep.

