# Supplementary material for: Androgen receptor regulates eIF5A2 expression and promotes prostate cancer metastasis via EMT
Source: Cell Death Discov. 2021 Dec 4;7:373. doi: 10.1038/s41420-021-00764-x (PMC8643356; doi:10.1038/s41420-021-00764-x)
Supplement: Supplementary file 1 — Supplementary Files [file 41420_2021_764_MOESM1_ESM.docx]

**Supplemental Figure legends**


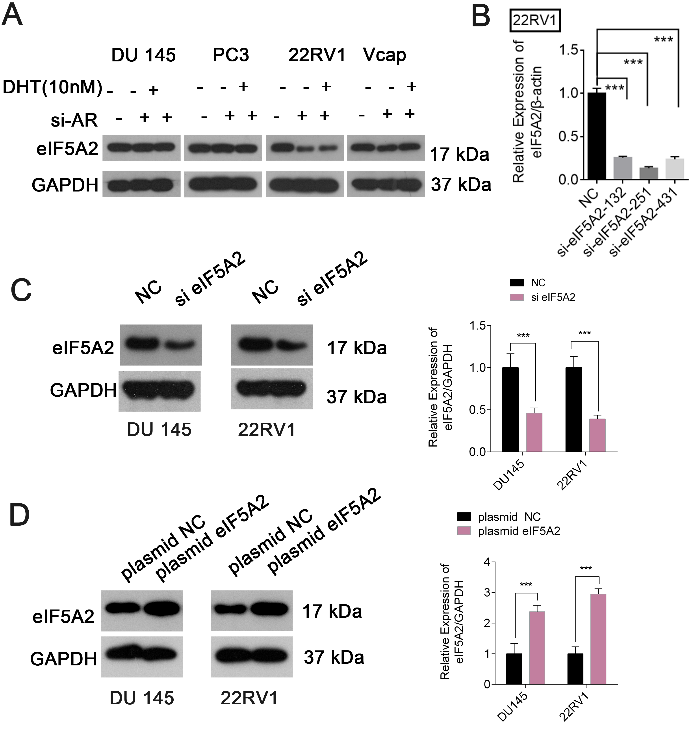


**Figure S1**

A. Western blot analysis of eIF5A2 expression after co-treatment with AR siRNA and DHT (10nM). B. The interference efficiency of eIF5A2was confirmed by qRT-PCR ***P < 0.001. C-D. Western blot showing eIF5A2 expression with or without transfection with eIF5A2 siRNA and eIF5A2 plasmid ***P < 0.001.


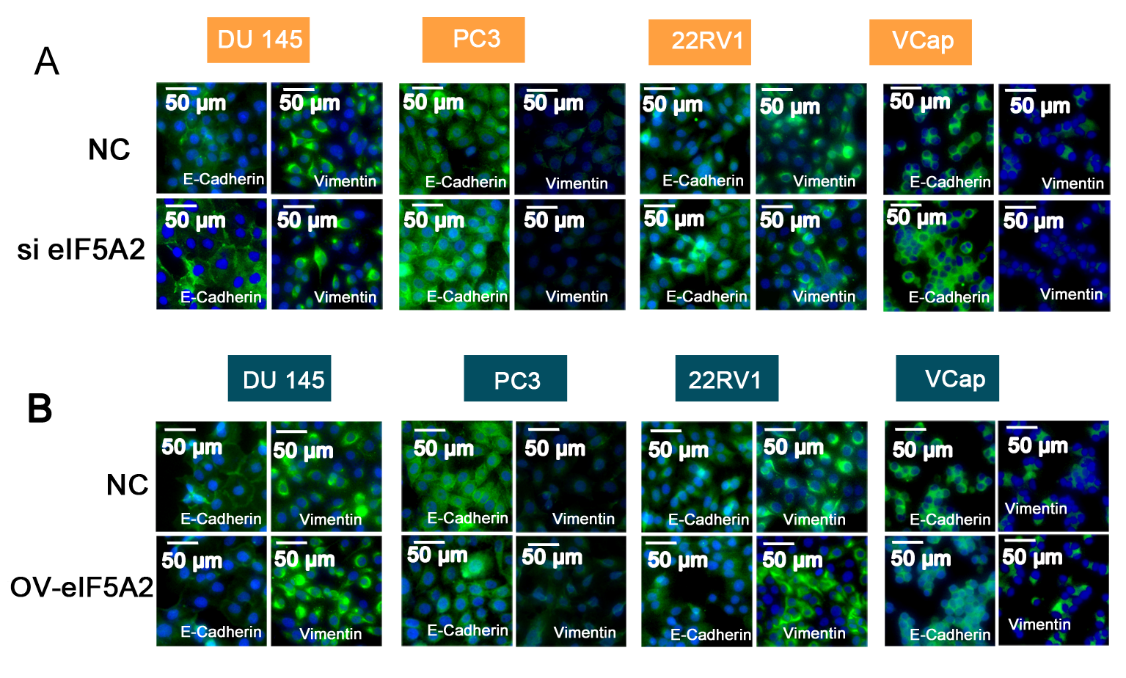


**Figure S2**

A-B. Immunofluorescence staining of E-cadherin and Vimentin in four PCa cell lines transfected with eIF5A2 siRNA, eIF5A2 plasmid, or negative control. (*P < 0.05, ***P* < 0.01, ****P* < 0.001 versus NC).


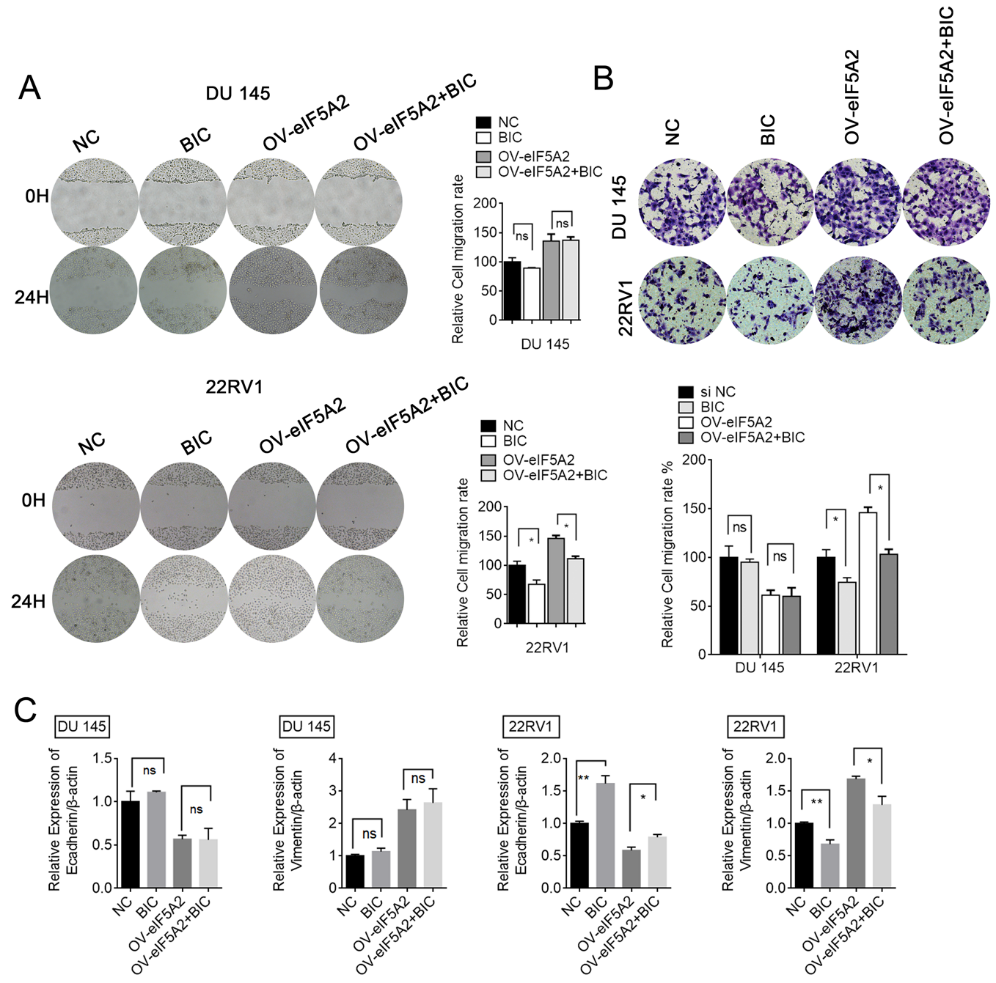


**Figure S3**

A. Migration and invasion capacity of DU145 and 22RV1 cells transfected with eIF5A2 plasmid and BIC. B. The EMT-related mRNA expression was examined following transfection with eIF5A2 plasmid with or without BIC treatment.


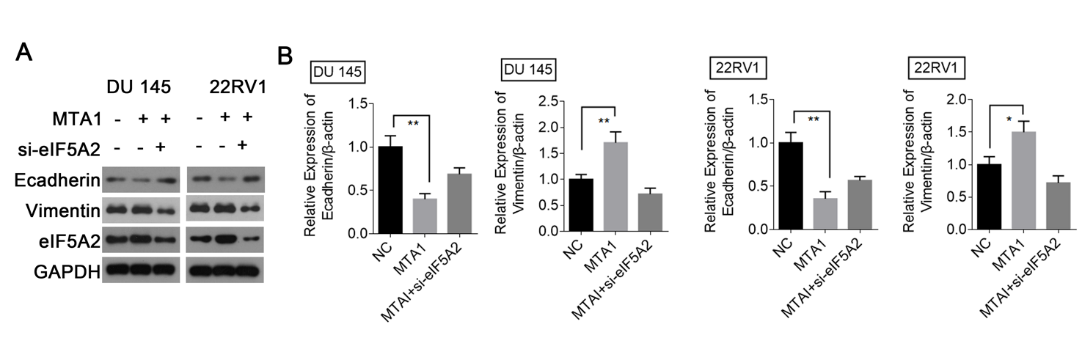


**Figure S4**

A. Western blot analysis of E-cadherin and Vimentin expression after transfection with MTA1 plasmid with or without eIF5A2 siRNA. B. qRT-PCR analysis of E-cadherin and Vimentin expression **P < 0.01.

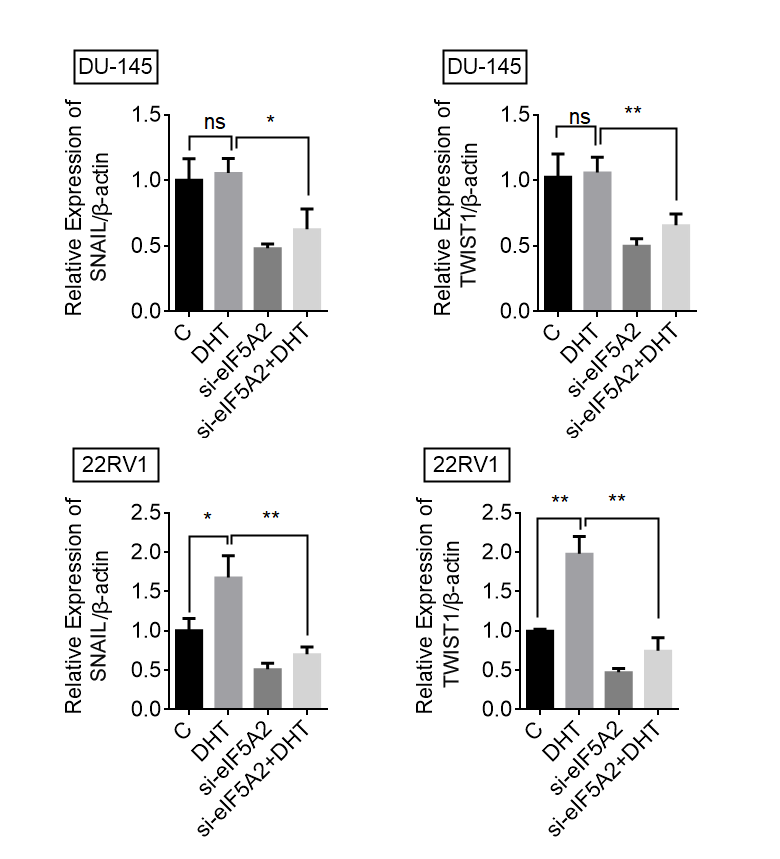


**Figure S5**

QRT-PCR analysis of TWIST1 and snail expression. *P < 0.05,**P < 0.01.
