# Supplementary material for: Androgen receptor regulates eIF5A2 expression and promotes prostate cancer metastasis via EMT
Source: Cell Death Discov. 2021 Dec 4;7:373. doi: 10.1038/s41420-021-00764-x (PMC8643356; doi:10.1038/s41420-021-00764-x)
Supplement: Supplementary file 2 — Authorship [file 41420_2021_764_MOESM2_ESM.pdf]

SPRINGER NATURE

Change of authorship request form - Journals (pre-acceptance)

Important information. Please read.

- This form should be used by authors to request any change in authorship (adding/deleting authors) including changes in corresponding authors. This form should not be used for name changes. Please fully complete all sections. Use black ink and block capitals and provide each author's full name with the given name first followed by the family name.
- By signing this declaration, all authors guarantee that the order of the authors are in accordance with their scientific contribution, if applicable as different conventions apply per discipline, and that only authors have been added who made a meaningful contribution to the work.
- Please note, in author collaborations where there is formal agreement for representing the collaboration, it is sufficient for the representative or legal guarantor (usually the corresponding author) to complete and sign the Authorship Change Form on behalf of all authors, next to the added/removed author(s). (Complete Section 3, followed by Section 6.)
  In author collaborations where there is no formal agreement for representing the collaboration and there are more than 10 authors, one may sign for all, provided the signer appends correspondence that attests that each of the authors have agreed to the change and the added/removed authors sign the form. (Complete Section 3, followed by Section 6.)
- Please note, we cannot investigate or mediate any authorship disputes. If you are unable to obtain agreement from all authors (including those who you wish to be removed) you must refer the matter to your institution(s) for investigation. Please inform us if you need to do this.
- If you are not able to return a fully completed form within **30 days** of the date that it was sent to the author requesting the change, we may have to withdraw your manuscript. We cannot publish manuscripts where authorship has not been agreed by all authors (including those who have been removed).
- Incomplete forms will be rejected.
- Please return/upload this form, fully completed, to the Journals Editorial Office. The Journal and/or Publisher will consider the information you have provided to decide whether to approve the proposed change in authorship. We may decide to contact your institution for more information or undertake a further investigation, if appropriate, before making a final decision.

扫描全能王 创建

SPRINGER NATURE

Change of authorship request form - Journals (pre-acceptance)

Section 1: Please provide the current title of manuscript

Manuscript ID no.: CDDISCOVERY-21-1998R

Title: Androgen receptor regulates eIF5A2 expression and promotes prostate cancer metastasis via EMT

Section 2: Please provide the previous authorship, in the order shown on the manuscript before the changes were introduced. Please indicate the corresponding author by adding (CA) behind the name.

	First name(s)	Family name	ORCID or SCOPUS id, if available
1st author	Yuancai	Zheng	
2nd author	Ping	Li	
3rd author	Hang	Huang	
4th author	Xueting	Ye	
5th author	Wei	Chen	
6th author	Guodong	Xu	
7th author	Fangyi	Zhang	
8th author			
9th author			
10th author			

Please use an additional sheet if there are more than 10 authors.

SPRINGER NATURE Springer Nature is one of the world's leading global research, educational and professional publishers, created in May 2015 through the combination of Nature Publishing Group, Palgrave Macmillan, Macmillan Education and Springer Science+Business Media.

扫描全能王 创建

SPRINGER NATURE

Change of authorship request form - Journals (pre-acceptance)

Section 3: Please provide a justification for change. Please use this section to explain your reasons for changing the authorship of your manuscript, e.g. what necessitated the change in authorship? Please refer to the (journal) policy pages for more information about authorship. Please explain why omitted authors were not originally included and/or why authors were removed on the submitted manuscript.

Section 4: Proposed new authorship. Please provide your new authorship list in the order you would like it to appear on the manuscript. Please indicate the corresponding author by adding (CA) behind the name. If the Corresponding Author has changed, please indicate the reason under section 3.

	First name(s)	Family name (this name will appear in full on the final publication and will be searchable in various abstract and indexing databases)	Affiliated institute	E-mail address
1st author	Yuancai	Zheng	The First Affiliated Hospital of Wenzhou Medical University	5552631@qq.com
2nd author	Ping	Li	The First Affiliated Hospital of Wenzhou Medical University	245706411@qq.com
3rd author	Hang	Huang	The First Affiliated Hospital of Wenzhou Medical University	huanghang2017@163.com
4th author	Xueting	Ye	The First Affiliated Hospital of Wenzhou Medical University	yexueting001@163.com
5th author	Wei	Chen	The First Affiliated Hospital of Wenzhou Medical University	wzmuchenwei@163.com
6th author	Guodong	Xu	The Affiliated Hospital, Ningbo Medical Center Lihuili Hospital, Ningbo University	13616577156@163.com
7th author	Fangyi	Zhang	The First Affiliated Hospital of Wenzhou Medical University	lzq640815@163.com
8th author				
9th author				
10th author				

Please use an additional sheet if there are more than 10 authors.

扫描全能王 创建

SPRINGER NATURE **Change of authorship request form - Journals** (pre-acceptance)

Section 5: Author contribution, Acknowledgement and Disclosures. Please use this section to provide a new disclosure statement and, if appropriate, acknowledge any contributors who have been removed as authors and ensure you state what contribution any new authors made (if applicable per the journal or book (series) policy). Please ensure these are updated in your manuscript - after approval of the change(s) - as our production department will not transfer the information in this form to your manuscript.

New acknowledgements:

Not applicable

New Disclosures (financial and non-financial interests, funding):

This study was supported by Zhejiang Provincial Natural Science Foundation of China (No. LQ20H050001 and LY20H160013), Wenzhou Science and Technology Project (Y20190066), Zhejiang Medical and Health Science and Technology Plan Project (2019KY456), The Public Welfare Technology Plan Project of Ningbo (No. 2019C50041), The Medical and Health Project Platform plan of Zhejiang Province (NO. 2019319634).
The authors declare that they have no competing interests.

New Author Contributions statement (if applicable per the journal policy):

Fangyi Zhang and Zhongqiu Lu conceptualized and planned the study; Yuancai Zheng and Ping Li performed the experiments; Hang Huang, Xueting Ye, Wei Chen, and Guodong Xu analyzed the data; Fangyi Zhang wrote the manuscript. All authors have read and approved the final version of the manuscript.

State 'Not applicable' if there are no new authors.

SPRINGER NATURE Springer Nature is one of the world's leading global research, educational and professional publishers, created in May 2015 through the combination of Nature Publishing Group, Palgrave Macmillan, Macmillan Education and Springer Science+Business Media.

扫描全能王 创建

SPRINGER NATURE Change of authorship request form - Journals (pre-acceptance)

Section 6: Declaration of agreement. *All* authors, unchanged, new and removed *must* sign this declaration.
(NB: Please print the form, (docu)-sign and return/upload a scanned copy. Please note that signatures that have been inserted as an image file are acceptable as long as it is handwritten. Typed names in the signature box are unacceptable.) *** Please delete as appropriate. Delete all of the bold if you were on the original authorship list and are remaining as an author.**

	First name	Family name		Signature	Date
1st author	Yuancai	Zheng	I agree to the proposed new authorship shown in section 4 **/and the addition/removal*of my name to the authorship list /and the proposed change in corresponding author**	Yuancai Zheng	15.Sep.2021
2nd author	Ping	Li	I agree to the proposed new authorship shown in section 4 **/and the addition/removal*of my name to the authorship list /and the proposed change in corresponding author**	Ping Li	15.Sep.2021
3rd author	Hang	Huang	I agree to the proposed new authorship shown in section 4 **/and the addition/removal*of my name to the authorship list /and the proposed change in corresponding author**	Hang Huang	15.Sep.2021
4th authors	Xueting	Ye	I agree to the proposed new authorship shown in section 4 **/and the addition/removal*of my name to the authorship list /and the proposed change in corresponding author**	Xueting Ye	15.sep.2021
5th author	Wei	Chen	I agree to the proposed new authorship shown in section 4 **/and the addition/removal*of my name to the authorship list /and the proposed change in corresponding author**	Wei chen	15.sep.2021
6th author	Guodong	Xu	I agree to the proposed new authorship shown in section 4 **/and the addition/removal*of my name to the authorship list /and the proposed change in corresponding author**	Guodong Xu	15.sep.2021
7th author	Fangyi	Zhang	I agree to the proposed new authorship shown in section 4 **/and the addition/removal*of my name to the authorship list /and the proposed change in corresponding author**	Fang yi Zhang	15.sep.2021

SPRINGER NATURE Springer Nature is one of the world's leading global research, educational and professional publishers, created in May 2015 through the combination of Nature Publishing Group, Palgrave Macmillan, Macmillan Education and Springer Science+Business Media.

扫描全能王 创建

SPRINGER NATURE Change of authorship request form - Journals (pre-acceptance)

	First name	Family name		Signature	Date
8th author			I agree to the proposed new authorship shown in section 4 /and the addition/removal*of my name to the authorship list /and the proposed change in corresponding author		
9th author			I agree to the proposed new authorship shown in section 4 /and the addition/removal*of my name to the authorship list /and the proposed change in corresponding author		
10th author			I agree to the proposed new authorship shown in section 4 /and the addition/removal*of my name to the authorship list /and the proposed change in corresponding author		

Please use an additional sheet if there are more than 10 authors.

In case of author collaborations with formal agreement:

	Name of consortium/consortia	First name	Family name		Signature	Date
Representative/legal guarantor				I agree to the proposed new authorship shown in section 4 /and the addition/removal*of my name to the authorship list /and the proposed change in corresponding author		

Both added/removed authors should complete the information in the first table under Section 6.

---- End of form ----

SPRINGER NATURE Springer Nature is one of the world's leading global research, educational and professional publishers, created in May 2015 through the combination of Nature Publishing Group, Palgrave Macmillan, Macmillan Education and Springer Science+Business Media.

扫描全能王 创建
